# Apollo’s Arrow: The Profound and Enduring Impact of Coronavirus on the Way We Live

**DOI:** 10.3201/eid2705.210381

**Published:** 2021-05

**Authors:** Nkuchia M. M’ikanatha, Christopher E. Carr

**Affiliations:** Pennsylvania Department of Health, Harrisburg, Pennsylvania, USA (N.M. M’ikanatha);; Georgia Institute of Technology, Atlanta, Georgia, USA (C.E. Carr)

**Keywords:** coronavirus disease, COVID-19, epidemiology, medical history, mental health, pandemics, psychology, public health, respiratory infections, SARS-CoV-2, viruses, severe acute respiratory syndrome coronavirus 2

In 1969, misplaced optimism led some to proclaim it time to “close the book on infectious diseases”; this prediction has been shattered by the emergence and reemergence of various human pathogens (*1*). *Apollo’s Arrow: The Profound and Enduring Impact of Coronavirus on the Way We Live* by Nicholas A. Christakis provides a real-time account of events that began in December 2019 when doctors confirmed the first cases of a disease caused by a pathogen later named severe acute respiratory syndrome coronavirus 2 (SARS-CoV-2) (Figure). Christakis, a physician, epidemiologist, and sociologist at Yale University, unravels how and why the emergence of SARS-CoV-2 cascaded into a human catastrophe.

**Figure:**
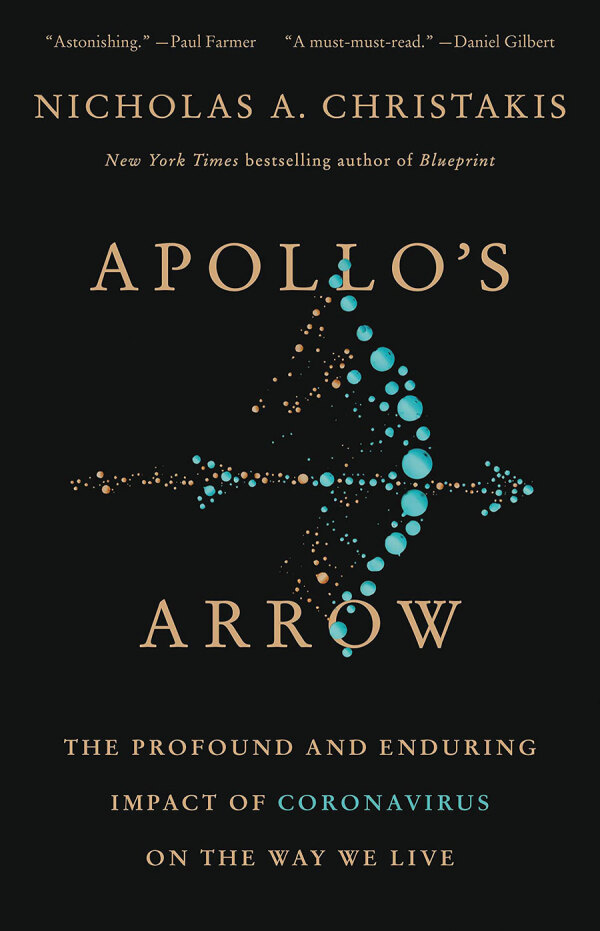
Apollo’s Arrow: The Profound and Enduring Impact of Coronavirus on the Way We Live

In the first 7 chapters of this well-researched and well-referenced book, focusing on events in the United States, Christakis describes the evolution of the coronavirus disease (COVID-19) pandemic and interprets these events in historical, human, and societal contexts. In Chapter 1, he uses epidemiological investigations of early cases to describe the onset of the pandemic. Christakis notes the unfortunate timing of the virus emerging during the leadup to the Lunar New Year celebration, when >3 billion trips are made within China and, while worldwide travel continued unabated, how the first confirmed COVID-19 case-patient was identified in the United States.

Christakis describes how using whole-genome sequencing to map SARS-CoV-2 variants spurred research and vaccine development. Contrasting the novel virus with previous human coronaviruses, Christakis uses analyses of social interactions to highlight the importance of host variation in superspreading. He writes: “SARS-CoV-2 has a positive mismatch period; this allows for asymptomatic transmission and makes traditional public health responses… very difficult.”

Christakis cites analogous examples of mask-wearing mandates in California during the 1918 Spanish flu pandemic to compare societal responses then and now. He illuminates how, despite the many social controversies around COVID-19 response, Americans overall have adopted social distancing measures with generosity, cooperation, and ingenuity. He also writes about the sacrifices made by healthcare and custodial workers who risked their lives during the first wave of COVID-19. Yet, Christakis reminds us that this personal altruism occurred while leaders even in wealthy countries failed to fully provide necessary protective equipment (*2*).

The book shines in its epidemiological and clinical observations and in descriptions of disproportionate illness and death among subpopulations in the United States. For example, Christakis highlights outbreaks of COVID-19 in meat processing plants with horrific working conditions. At other times he seems overly optimistic, such as when he suggests that a lasting impact of COVID-19 may be “respect for science and expertise, even when it leads to people taking actions they would rather avoid.” Christakis highlights the successful implementation of COVID-19 control measures in China and New Zealand and notes the United States’ ability to spend trillions of dollars on mitigation measures, including vaccine development. 

*Apollos’s Arrow* balances the gloom of the pandemic period with insights from the past, accounts of hands-on patient care, and population-level observations. We were also delighted with the author’s engaging narration in the audiobook. This book will interest anyone questioning why, despite impressive advances in biological sciences, COVID-19 has evolved into an unimaginable global catastrophe, resulting in >119.6 million reported illnesses and >2.6 million deaths by mid-March 2021 (*3*).
